# Interventional embolization therapy for anterior choroidal artery aneurysms

**DOI:** 10.1097/MD.0000000000042923

**Published:** 2025-10-31

**Authors:** Xiyong Hu, Weina Xu, Wei Li, Lei Xu

**Affiliations:** a Department of Neurosurgery, Weifang People’s Hospital, Shandong Second Medical University, Weifang, China; b Department of Neurology, Weifang Traditional Chinese Medicine Hospital, Weifang, China.

**Keywords:** anterior choroidal artery, anterior choroidal artery aneurysm, interventional embolization therapy

## Abstract

This study aims to explore the clinical efficacy and surgical techniques of interventional embolization therapy for anterior choroidal artery aneurysms (AchANs). We retrospectively collected clinical data from 25 patients with AchANs treated with interventional therapy in our hospital from October 2021 to May 2024, including 16 cases with unruptured aneurysms and 9 cases with ruptured aneurysms. All 25 cases of aneurysms had relatively or absolutely wide necks and were treated with stent assisted coil embolization. All 25 patients achieved subtotal embolism. During interventional embolization surgery, 3 cases experienced choroidal artery thrombosis or spasm, and were treated with tirofiban simultaneously. Due to cerebral infarction, all the 3 patients had limited limb mobility on 1 side. After active treatment for cerebral infarction, one of them showed improvement in symptoms. Flexibly utilizing intervention techniques to ensure the patency of the anterior choroidal artery is crucial for interventional embolization of AchANs.

## 1. Introduction

Anterior choroidal artery aneurysm (AchAN) accounts for 2% to 5% of intracranial aneurysms.^[1]^ Due to the particularity of the anterior choroidal artery (AchA), it belongs to the terminal branch of the internal carotid artery, with few collateral circulation compensation, thin diameter, long stroke, and supplies blood to important structures such as the optic tract, cerebral peduncle, lateral geniculate body, and posterior limb of internal capsule. So, the AchA spasm or blockage during the surgical process will lead to serious complications.^[2]^ Therefore, taking a series of measures to ensure the patency of the AchA is the key to the surgery. Retrospective collection and analysis of the data from patients with AchAN treated with interventional therapy at our center. The summary of some surgical techniques is as follows, aiming to summarize clinical experience and reduce postoperative complications of interventional embolization for AchANs.

## 2. Materials and methods

### 2.1. Study design and patient selection

We retrospectively collected data from 25 patients with AchAN who underwent interventional treatment in our hospital from October 2021 to May 2024. Among them, there were 14 males and 11 females, with an age of (56.5 ± 4.7) years; 16 cases with unruptured aneurysms and 9 cases with ruptured aneurysms; all 25 cases of aneurysms had relatively or absolutely wide necks and were treated with stent assisted coil embolization; 22 cases were combined with hypertension; 12 cases with diabetes.

Inclusion criteria: Confirmed by cerebral angiography, the AchAN has ruptured or at risk of rupture; Hunt Hess rating ≤2; Patients with severe stenosis, obvious atherosclerosis, and severe underlying diseases were excluded; Surgical treatment has been approved and signed by the patient themselves, their family members, or their guardians.

### 2.2. Operation

**Figure 1.**
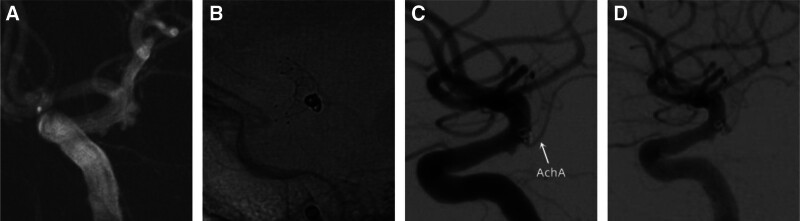
“Lantern” technique—left anterior choroidal artery aneurysm stent assisted coil embolization. (A) Cerebral angiography confirmed a left anterior choroidal artery aneurysm, approximately 2 × 4 mm in size, with the apex pointing backwards and irregular in shape, posing a risk of rupture and bleeding; (B) The “lantern” technique allows the stent to protrude into the aneurysm, resulting in a subtotal embolization of the aneurysm; (C) Postoperative angiography showed that the anterior choroidal artery and intracranial artery were unobstructed; (D) Postoperative follow-up cerebral angiography showed no recurrence of the aneurysm, and the anterior choroidal artery was unobstructed.

After successful general anesthesia with endotracheal intubation, the patient is placed in a supine position and the skin in both inguinal areas is disinfected. Whole body heparinization, the intermediate catheter is placed in the C4 segment of the internal carotid artery under the guidance of a loach guidewire. Select the working position and insert the stent microcatheter into the M1 segment of the middle cerebral artery under the guidance of a micro-guide wire. The embolization microcatheter is inserted into the AchAN under the guidance of a micro-guide wire. Choose the spring coil to form a basket. The stent is partially released and covers the neck of the aneurysm through the stent microcatheter. Adjust and continue to release the spring coil, and the imaging confirms that the aneurysm is no longer visible. Release the stent completely. Further imaging confirmed that there is no visualization of the AchAN, and the parent artery and AchA are unobstructed. Dyna CT do not show any signs of cerebral hemorrhage, so the surgery is terminated (Fig. 1).

### 2.3. Observation indicators and evaluation

According to the modified Rankin scale, the prognosis of patients is evaluated 1 month after surgery. A score of 0 to 2 indicates good prognosis, 3 to 5 indicates poor prognosis, and 6 indicates patient death.

Evaluate the postoperative cerebral infarction and cerebral hemorrhage in patients based on their perioperative clinical symptoms and imaging data (cranial CT, MRI).

By observing the blood flow velocity inside the stent and in the arteries during surgery, the incidence of thrombosis can be determined.

The degree of embolization of an aneurysm is determined by measuring the proportion of unfilled areas in the aneurysm cavity using cerebral angiography.^[3]^ Embolization of 95% to 100% is considered complete, and 80% to 95% is considered subtotal.

### 2.4. Follow-up

The patients were followed up 6 to 12 months after surgery, and the prognosis was evaluated using the modified Rankin scale. All 25 patients underwent cerebral angiography reexamination, and no recurrence was found in the aneurysms. Two cases had occlusion of the AchA, leaving behind symptoms of limb hemiplegia.

## 3. Results

All 25 patients achieved subtotal embolism. During interventional embolization surgery, 3 cases experienced choroidal artery thrombosis or spasm, and were treated with tirofiban simultaneously. Due to cerebral infarction, all the 3 patients had limited limb mobility on 1 side. After active treatment for cerebral infarction, one of them showed improvement in symptoms.

## 4. Discussion

AchAN is relatively rare in clinical practice, with a small body and a wide neck.^[1]^ According to the position relationship between the aneurysm and the parent artery, it can be divided into 3 types: type A, type J, and type I. Among them, the type J is the most common (47%).^[4]^ Due to the fact that the AchA is the terminal artery of the internal carotid artery, slender with an average diameter of 0.75 to 1.20 mm,^[5]^ and originates from the posterior wall of the internal carotid artery, clipping of intracranial aneurysm may lead to vascular spasm, misidentification, and occlusion of the AchA.^[6]^ Compared with this, interventional embolization therapy can clearly distinguish the position relationship between the AchA and the aneurysm, and better protect the AchA.^[1]^ This study explores the interventional treatment of J-type AchAN. Due to the fact that the AchA supplies blood to important brain tissues such as the thalamus, ischemia is the most common and serious surgical complication, including cerebral vasospasm and arterial thrombosis, requiring the surgeon to have extensive experience in embolization.

**Figure 2.**
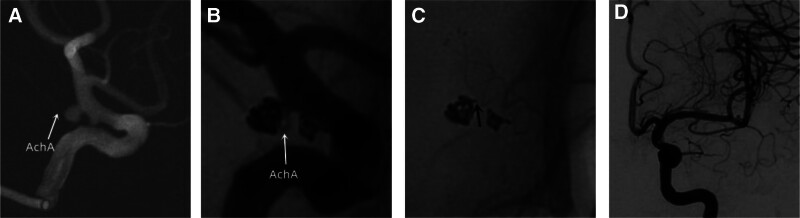
Stent assisted coil embolization for left anterior choroidal artery aneurysm and posterior communicating artery aneurysm. (A) Cerebral angiography confirmed the presence of aneurysms in the left anterior choroidal artery and left posterior communicating artery. The size of the aneurysm in the anterior choroidal artery is approximately 3 × 4 mm, with the apex pointing backwards and an irregular shape, posing a risk of rupture; (B, C) Subtle embolization of aneurysms, using a technique similar to the “suspended scaffold,” with a spring coil supported on the stent to ensure the patency of the anterior choroidal artery (white arrow pointing to the postoperative unobstructed anterior choroidal artery, black arrow pointing to a spring coil ring structure supported on the stent); (D) Postoperative angiography showed good visualization of all branches of the internal carotid artery.

With the development of medical imaging, more and more intracranial unruptured small aneurysms have been discovered,^[7]^ and AchAN are mostly small aneurysms with high rupture and bleeding rates.^[8,9]^ Discovering high-risk AchAN and early surgical intervention has significant clinical significance. Retrospective summary and analysis of clinical experience in interventional embolization of AchAN in our hospital: First, most AchANs are small in size and have a wide neck, and the stent “lantern” technique can be used to narrow the aneurysm neck to avoid coil detachment; Increase the metal coverage at the neck of the aneurysm, enhance blood flow guidance, change hemodynamics, and avoid aneurysm recurrence; This type of stent often uses woven stents, such as LVIS stent^[10]^ (Fig. 1); Second, the spring coil support technique repeatedly adjusts the spring coil into a basket during the process of arterial aneurysm embolization, so that 1 or more rings of the spring coil are supported on the stent, which is beneficial for the subsequent spring coil to form a basket. Its purpose is to ensure the patency of the AchA (black arrow in Fig. 2); Third, to avoid the displacement of the coil affecting the blood flow of the AchA, a microcatheter can be super selected into the AchA to assist in embolization of the aneurysm before embolization. However, having 3 microcatheters during surgery increases the difficulty of surgical operation and the risk of thrombosis formation; Fourth, use the flow diverter: For complex aneurysms and blood blister-like aneurysms, the flow diverter is a safe and effective treatment option.^[11]^ Its mechanism is to slow down blood flow and cause thrombosis in the aneurysm, but due to the special nature of the AchA, once blood flow stagnates, it will lead to serious complications. Fifth, research has shown that ischemic complications after stent assisted embolization of AchAN are related to factors such as aneurysm size,^[12]^ embolization density, and stent selection. Avoid excessive embolization causing stenosis or occlusion of the AchA. All patients in this study had subtotal embolism. In this study, patients were treated with stent semi release technique and flexible use of interventional embolization techniques such as “lantern” technique and spring coil support technique. There were 3 patients who experienced symptoms of cerebral infarction after surgery. Among them, 2 cases were considered to be caused by acute thrombosis of the AchA, and 1 case was believed to be caused by subarachnoid hemorrhage stimulating spasm of the AchA. After active medication treatment, 1 patient’s symptoms were completely relieved.

To avoid occlusion of the AchA during surgery: First, prior to embolization, 3D scanning is performed to clarify the positional relationship between the aneurysm neck and the AchA, as well as the internal carotid artery. The optimal working position is selected, and embolization treatment is performed at the maximum magnification; Second, during the embolization process, the relationship between the coil and the neck of the aneurysm should be observed constantly through imaging, and release the coil once the AchA is confirmed to be unobstructed; Third, avoiding excessive embolism, the recurrence rate of subtotal embolism is relatively low, but the safety rate is high.^[1]^

If occlusion of the AchA is found during surgery, the first step is to determine whether it is caused by the coil, and if necessary, retrieve the coil and refill it; If it is confirmed that there is thrombosis in the AchA, immediate treatment with tirofiban should be given, and intravenous infusion should be continued according to body weight. Most of the AchAs will be reopened; For patients whose arteries have not reopened, follow the treatment principles of acute cerebral infarction immediately, adding antiplatelet drugs, raising blood pressure, and increasing blood flow to reduce the area of cerebral infarction.

In summary, the key to interventional embolization therapy for AchAN is to ensure the patency of the AchA on the basis of embolization of the aneurysm. Proficient use of spring coil and stent assisted techniques, flexible handling of intraoperative emergencies, and reduction of postoperative cerebral infarction events are necessary to ensure the quality of life of patients after surgery.

## Author contributions

**Data curation:** Wei Li.

**Project administration:** Weina Xu.

**Writing – review & editing:** Lei Xu.

**Writing – original draft:** Xiyong Hu.
